# Delineation of the Feline Hippocampal Formation: A Comparison of Magnetic Resonance Images With Anatomic Slices

**DOI:** 10.3389/fvets.2019.00358

**Published:** 2019-11-08

**Authors:** Isabella Gruber, Sibylle Kneissl, Alexander Probst, Akos Pakozdy

**Affiliations:** ^1^Internal Medicine Small Animals, University of Veterinary Medicine, Vienna, Austria; ^2^Diagnostic Imaging, University of Veterinary Medicine, Vienna, Austria; ^3^Institute of Topographic Anatomy, University of Veterinary Medicine, Vienna, Austria

**Keywords:** feline, hippocampal formation, anatomical borders, magnetic resonance imaging, anatomic slices

## Abstract

The hippocampal formation (HF) is a relevant brain structure that is involved in several neurological and psychiatric diseases. In cats, structural changes of the HF are associated with epilepsy. The knowledge of a detailed anatomy of this brain region may lead to the accurate diagnosis and development of better therapies. There are, however, discrepancies among the research findings, which may be due to different definitions being used, according to anatomical guidelines and boundaries, as well as different magnetic resonance (MR) protocols. The aim of this study is to evaluate the anatomical borders of the HF on transverse MR images and the correlated anatomic sections in three cats. The boundaries of the HF were mostly visible in the formalin fixed anatomic sections, except in the areas where the hippocampus proper exchanges into the subicular complex. Also, the delineation of the anteroventral part and the latero-caudal borders of the HF were not clearly defined. Based on our preliminary results these problems are reinforced on MR images, and further histological and anatomical research must be done to find a way to delineate these neurological structures accurately.

## Introduction

**Figure 1:**
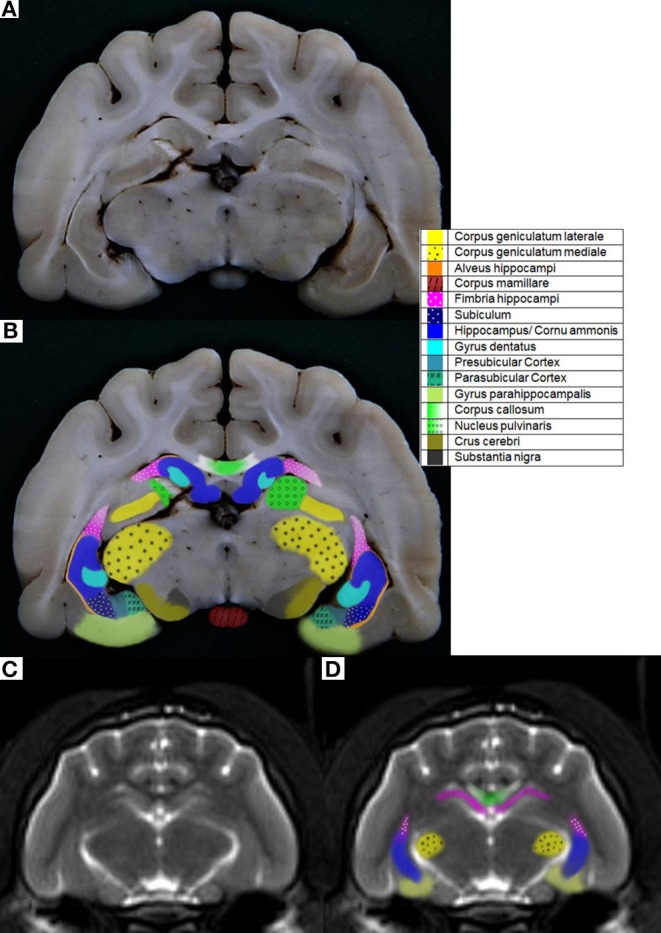
**(A)** Cat 1 slice 7 **(B)** colored with pre- and parasubicular Cortex **(C)** MR image cat 1 brain slice 7 T2-weighted according to Rusbridge et al. (17) **(D)** colored.

The hippocampal formation (HF) is a relevant brain structure that is involved in many neurological and psychiatric diseases. The main components of the HF are the subiculum, the hippocampus proper (also called cornu ammonis, CA), and the dentate gyrus (DG) (1–13). These components are highly folded into and around each other. The HF is located in the medial surface of the temporal lobe, along the floor and medial wall of the temporal horn of the lateral ventricle (2, 10, 13). The ventricular surface of the HF is covered by a thin layer of white fibers, the alveus hippocampi (1, 5, 10, 13, 14). The subiculum is the transition region from the CA to the parahippocampal gyrus (12). It would be better named as the subicular complex, because it can be divided into subregions. In cats, a presubiculum and parasubiculum can be differentiated (15, 16) (Figure 1B).

In cats some diseases such as epilepsy are associated with structural changes of the HF, especially feline temporal lobe epilepsy (FTLE) (18). Cats suffering from FTLE often show an affected HF with loss of normal internal architecture, altered signal intensity and decreased volume in magnetic resonance imaging (MRI) (18–27). The knowledge of these underlying pathologies can indicate the functions of particular brain structures, which could lead to a better understanding of this disease/FTLE and prognosis (22, 25). MR imaging is an essential tool for the ante-mortem diagnosis of structural changes in the brain. MR imaging based hippocampal volumetry is a useful method to measure the anatomic size of the hippocampus in human patients (25, 28–31). The use of MR imaging to describe HF volume has also been used in dogs and cats (10, 13, 32). There is conflicting evidence of HF volume in the literature, which may be due to the use of different methods of volume measurement, different MR imaging protocols and different definitions according to anatomical guidelines (30). The aim of this study was to evaluate the anatomical borders of the HF in cats, based on transversal 1.5 T MR imaging, compared with transversal formalin fixed brain slices.

## Materials and Methods

### Animals

For the comparison, MR images and formalin fixed slices from three different male neutered European shorthair cats were used. None of these cats had a history of neurological disease. The cats were all between 10 and 13 years old and were euthanized due to serious non-intracranial illness at the owner‘s requests. They were immediately transferred into a cooling chamber (4°C) and imaged within 20 h after euthanasia.

### MRI

All cats were placed in sternal recumbency on the table of the magnetic resonance unit (Magnetom espree 1.5 T, Siemens Healthcare, Erlangen, Germany) The heads were placed and arranged into a 15-channel knee coil. Images obtained in sagittal, transverse, dorsal, or standardized oblique planes according to the literature (Table 1), were provided by the University Clinic for Diagnostic Imaging. According to Milne et al. (10), Mizoguchi et al. (24), and Rusbridge et al. (17) T2-weighted sequences in sagittal orientations were made, to enable the identification of the long axis of the hippocampus and dorsal, which was orientated perpendicular to the long axis of the hippocampus and transverse, parallel to the long axis of the hippocampus. Also, Fluid-Attenuated-Inversion-Recovery (FLAIR) sequences in dorsal and transverse planes, as well as a dorsal and oblique dorsal T1-weighted sequences were created. The imaging parameters varied throughout the sequences (Table 2). Transversal orientation is recommended in all protocols and is routinely used in the clinic every day; this was also used because the HF is well-recognized on transversal images. For this study, the HF was evaluated on transverse anatomical specimens and transversal MR images.

**Table 1:** Planned and performed (*) sequences according to the literature.

**MR Sequences according to Rusbridge et al. (17)**
1. sagittal T2- weighted sequence* (to identify the long axis of the hippocampus)
2. dorsal T2- weighted sequence* (perpendicular to the long axis of the HF)
3. transverse T2- weighted sequence* (parallel to the long axis of the HF)
4. dorsal FLAIR sequence (perpendicular to the long axis of the HF)
5. transverse FLAIR sequence* (parallel to the long axis of the HF)
6. dorsal T1- weighted sequence* (perpendicular to long axis of the HF)
**According to Milne et al**. **(****10****)**
7. Oblique dorsal T1-weighted sequence* (perpendicular to the long axis of the HF)
8. sagittal T2-weighted sequence*
9. transverse T2-weighted sequence* (perpendicular to the skull base)
10. transverse FLAIR sequence
**According to Mizoguchi et al**. **(****24****)**
11. FSE 3D T2-weighted Cube images
12. transverse T1-weighted FLAIR sequence
13. transverse T2-weighted sequence*
14. transverse T2-weighted FLAIR sequence*
15. post contrast transverse T1-weighted FLAIR sequence

*For this study however, the HF was only evaluated on transverse anatomical specimens and transversal MR images*.

**Table 2:** Parameters from the performed sequences.

**No**.	**Sequence**	**Device**	**2D/3D**		**TR**	**TE**	**Flip angle**	**NEX**	**Slice thickness**	**Interslice gap**	**Field of view**	**Matrix**
**1**.	T2 3D sag	Siemens espree	3D	TSE	3000.0	388.0	120	2	0.8	0.8	157*180	256*226
**2**.	T2 3D dor	Siemens espree	3D	TSE	3000.0	388.0	120	2	0.8	0.8	157*180	256*226
**3**.	T2 tra oblique	Siemens espree	2D	SE	5370.0	111.0	150	4	2.5	0.5	130*130	256*205
**4**.	FLAIR tra	Siemens espree	2D	TSE-TIR-FS	8500.0	79.0	150	1	2.5	0.5	129*129	192*192
**5**.	T1 3D dor	Siemens espree	3D	GR/IR	1720.0	5.5	15	1	0.9	0.9	170*170	256*246
**6**.	T1 3D oblique dor (Milne)	Siemens espree	3D	GR/IR	1720.0	5.5	15	1	0.9	0.9	170*170	256*246
	T2 sag (see 1.)	Siemens espree										
**7**.	T2 tra	Siemens espree	2D	TSE	5370.0	111.0	150	4	2.5	0.5	130*130	256*205
	T2 3D dor (see 2.)	Siemens espree										
	T2	Siemens espree										
	Flair	Siemens espree										

*Field of view in cm x cm, Flip angle in °, Interslice gap in mm, Matrix in pixels, NEX (number of acquisitions), TE (echo time) in ms, TR (repetition time) in ms, TSE (turbo spine echo), SE (spine echo), slice thickness in mm*.

### Formalin Fixation and Slicing

After the magnetic resonance imaging, the brains were immediately removed, fixed in 4% formalin solution and cut into two to 3 mm thick transverse slices, perpendicular to the long axis of the fissura longitudinalis cerebri (Figures S1, S2).

### Picture Processing

These slices were photographed with the photo-editing program GIMP 2 and as many visible anatomical structures as possible were delineated; these anatomical structures were named following the Nomina Anatomica Veterinaria (33) (NAV) and the Illustrated Veterinary Anatomical Nomenclature (34) with the help of Winkler and Potter (35) and Brainmaps (36). These structures were also colored according to these sources. The different magnetic resonance images were compared with formalin fixed slices and the stained pictures of the formalin fixed slices from cat brains (Figures S3–S6).

The same person did the imaging, the delineation, and review of the anatomical structures on formalin section and images. A senior neuroanatomist (A. Probst) and a senior neuroradiologist (S.K.) performed together with a senior neurologist (A. Pakozdy) the study.

## Results

Based on the NAV (37) terminology, the following structures were found on the formalin fixed slices: hippocampus proper/CA, dentate gyrus, alveus hippocampi, subiculum, parahippocampal gyrus, fimbria hippocampi, and the fornix. Furthermore, the fasciola cinerea, corpus geniculatum laterale and mediale, corpus amygdaloideum, nucleus ruber, nucleus caudatus, corpus mamillare, brachium colliculi caudalis, tractus mamillothalamicus, tractus opticus, claustrum, corpus callosum, stria terminalis, the crus cerebri with the adjacent substantia nigra, and the putamen could also be delineated on the anatomical specimens, but these structures could not be accurately found on the MR images. The delineation of the HF was mostly possible on transverse anatomical specimens, except that the boundaries of the ventral HF were not completely clearly traceable, especially in the slides where the hippocampus proper exchanges into the subicular complex; additionally, the differentiation of the presubiculum, parasubiculum and subiculum, and the exchanges into the parahippocampal gyrus were not completely clear (Figure S3). On the MR images, fewer anatomical brain structures could be seen, and the delineation was less accurate. The following structures were colored on the MR images: the HF, corpus amygdaloideum, corpus callosum, parahippocampal gyrus, fimbria hippocampi, and the fornix. The differentiation between the hippocampus proper, the dentate gyrus and alveus, as well as the presubiculum, parasubiculum, and subiculum, was not possible (Figures S3–S6).

In the caption, the opacity of the colors were 100%. In contrast to this, the opacity of the colors in the pictures was elected to be just 60% for better illustration (Figure 1).

## Discussion

The identification, delineation, and measurement of the HF are very important due to its involvement in many diseases. Throughout the literature, different MR imaging protocols were used for examination, and, depending on the author, the result of which sequence the HF was best delineated varied. Several authors, like Pantel et al. (25), Francis (32), Leigh et al. (38), Gray-Edwards et al. (39), and Przyborowska et al. (40), used and recommended T2-weighted MR images in cats. Kuwabara et al. (41) used T2-weighted and also T1-weighted MR sequences in their protocol for dogs, but their hippocampal volume measurement was made on the basis of the T2-weighted images, because the delineation was easier. Only a few authors recommended and used other sequences for examination of the HF (5, 12, 42). This matches the findings of this study, as most anatomical structures were best visible in the T2-weighted sequences. Also, the contrast was better in these MR images and so the borders of the HF were better delineated. An option for a better view of the HF would be to use a T2-inverted sequence, which was developed recently and needs to be investigated in the future. However, it should be noted that this is just for the delineation of the HF, and for clinical use it will be recommended to follow a protocol with at least additional FLAIR and T1-weighted sequences (43, 44).

**Figure 2:**
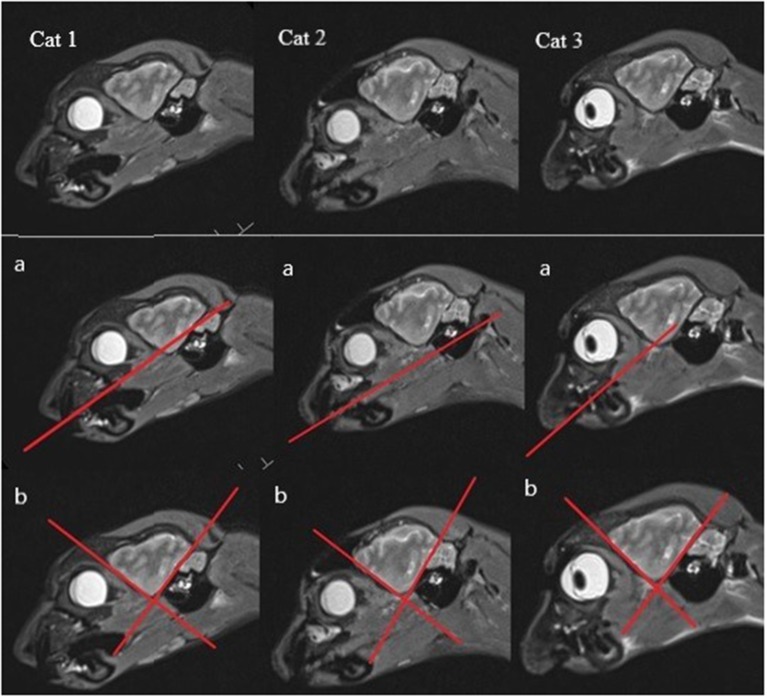
Anatomical variability shown on the sagittal MR images (with most of the HF visible) of each cat (a) with the hard palate singed in (b), one line marking the rostroventral brain contour, and the other a line following the tentorium cerebelli osseum, showing the different angles between these lines and thus the anatomical variability.

There have also been some problems with orientating the diverse MR imaging sequences according to the literature. For example, in Rusbridge et al. (17), the sagittal T2-weighted sequence should be orientated along the long axis of the HF, but since this is a curved structure, there are many possibilities for the orientation of the sequence. The instructions of Milne et al. (10) were to put the orientation of the plane for the transverse T2-weighted sequence perpendicular to the skull base, which seemed to have less variability in the orientation angle. In the research findings of other authors, the transverse MR sequences were orientated perpendicular to the hard palate, which seemed to be a good anatomic fixation point, but it was found that there is a possible range of 15 degrees (38, 40, 44, 45). Other authors have not included a detailed description of the orientation in their work. Therefore, to minimize this source of inaccuracy and to standardize the protocol for examination of the HF as well as avoiding large variability in orientation of the planes, concrete anatomical landmarks should be found. Another option would be to create a gauge with concrete anatomical landmarks and a line that shows how to orientate the plane. These ideas for better traceability will not be easily implemented due to individual anatomic variability (Figure 2).

For veterinarian patients, 0.2 T to 1.5 T MR imaging units are widely used (45–47). Some authors have also compared their findings in high magnetic field images with those in low magnetic field images (32, 40, 45, 46). The small size of the HF in animals and the differences in contrast between gray and white matter, compared to humans, may mean that a higher magnetic field strength will enable better delineation, especially in the ventral part, of the HF from the amygdala (10, 30, 44). However, in comparison to the improvement of 7 T images in human medicine, in veterinary medicine 7 T images do not offer a significant upgrade in image quality, which is partly a result of increased susceptibility artifacts and a lack of available imaging protocols. On the other hand, the high costs, increased acoustic noise and higher health risks make it unrealistic to use 7 T units in ordinary clinics or for regular investigations of the HF (32, 45, 46). The MR images in this investigation were made with a 1.5 T magnetic resonance unit, which seems to be the gold standard for MR imaging of the feline brain. The HF was visible, and, for a rough estimate or lesion search, it would be sufficient, but the borders can only be distinguished with moderate accuracy throughout the whole structure. A better delineation of the HF in MR images could be achieved by using MR imaging units with a higher magnetic field strength and therefore better image quality.

Among the literature, there is also some discrepancy as to which anatomical structures are contained within the HF. A few authors only include the DG and the CA in the HF (12, 41), but most authors define the components of the HF as the DG, CA, and the subiculum (10, 13, 32, 48, 49). Some also include the alveus and the fimbria (8, 50–53). Regarding their functions, connections, and afferent and efferent pathways of the individual structures, it would make sense to include the DG, CA, subicular complex, alveus and fimbria to the HF (11, 49). Furthermore, the identifiability of each anatomical structure, most notably of the alveus especially in the MR images, is restricted (Figure 1A).

**Figure 3:**
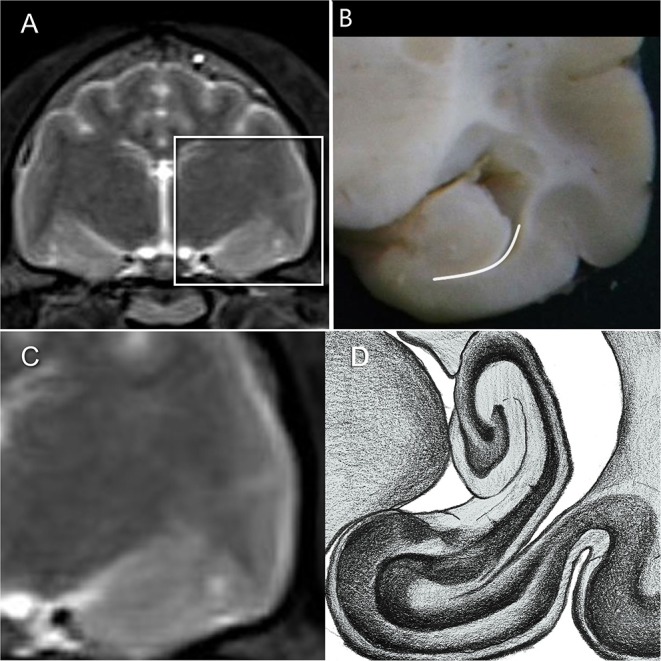
**(A,C)** HF on T2-weighted MR image **(B)** HF with the suggested border (dashed line) between the subicular complex and the gyrus parahippocampalis according to Mu et al. (52) **(D)** own reproduction of the HF after Brainmaps (36) and Winkler and Potter (35).

There are several definitions of the boundaries of the HF. In veterinarian medicine, the delineation of the HF is mostly described as being done through the CSF and the alveus in the rostromedial and lateral parts of the HF (10, 13). Delineation with the aid of the CSF, even in high resolution MR imaging, is rarely possible and this can also be seen in the results from our study, because in the ventricular system of healthy cats it is very small (13) (Figure 1A). Furthermore, delineation of the alveus is difficult and only possible in high resolution MR images; in the opinion of some authors, the alveus is most evident in the planes perpendicular to the long axis of the HF in dogs (10). The boundaries of the HF were mostly visible in the formalin fixed slices, except from the areas where the hippocampus proper exchanges into the subicular complex and then exchanges into the parahippocampal gyrus (Figure 1A). These boundaries have been insufficiently defined until now, especially for companion animals and using MR imaging, but it is not possible to find a macroscopically clear delineation of these structures (Figures 1C,D). Moreover, the delineation of the anteroventral part of the HF from the amygdala is not fully clear (10, 32, 41). The lateral caudal borders of the HF could be difficult to distinguish because of the adjacent thalamus (10). These problems are reinforced in the MR images. Though there is a good white-gray matter contrast in the T2-weighted MR images, the cell structure is even less visible (Figures 1C,D). Some authors who made volumetric measurements of the HF tried to make their results reproducible by defining a line fixed on anatomical structures that are clearly visible for the boundary of the subicular complex to the parahippocampal gyrus. Mu et al. (52) defined the boundary between the subiculum and parahippocampal gyrus by a line in continuation with the inferior border of the subiculum (Figures 3A–D). Milne et al. (10) defined the boundary between the same structures in the oblique dorsal plane as a line perpendicular to the tangent of the cortex at the apex of the cortex as it curved caudomedially. Gardini et al. (5) tried to quantify the HF *via* linear measurements and by creating a ratio between the height of the brain and the HF height. These methods seem to be a better solution to define a rough boundary and to obtain comparable results for the volumetric measurement (Figures 3A–D). However, for exact delineation, further histological and anatomical investigations are necessary.

For this study, only transverse formalin fixed slices were used, which is a limitation, but to slice the brain optimally in a transversal, dorsal, or sagittal plane, other methods are needed and the development of these would be beyond the scope of this work. Consequently, only transverse MR images were used in different sequences for comparison and delineation. A further limitation is that only three cats were included, and these were only examined postmortem, which could have caused a certain degree of blurring and lack of definition in the MR imaging.

## Conclusion

Based on our preliminary results the boundaries of the HF in routine 1.5 T MR images are not completely traceable, as delineation is difficult or even impossible, especially in the ventral portion. Consequently, volumetry currently has a high bias risk. Therefore, there is a need to determine a better method to delineate these structures before a volumetric MR imaging-based measurement of the HF could be accepted as accurate.

## Data Availability Statement

The datasets generated for this study are available on request to the corresponding author.

## Ethics Statement

No ethical approval needed for the use of cadaver waste material according to the good Scientific Practice Guidelines of the University of Veterinary Medicine, Vienna and applicable institutional and national guidelines and regulations.

## Author Contributions

All authors listed have made a substantial, direct and intellectual contribution to the work, and approved it for publication.

### Conflict of Interest

The authors declare that the research was conducted in the absence of any commercial or financial relationships that could be construed as a potential conflict of interest. The reviewer DH declared a past co-authorship with one of the authors AP to the handling Editor.

## Supplementary Material

Figure S1 Brain after the formalin fixation.

Figure S2 Overview over the produced slices.

Figure S3 **(A)** Cat 1 slice 8 **(B)** colored **(C)** colored with pre- and parasubicular Cortex **(D)** Brainmaps (36).

Figure S4 MR image cat 1 brain slice 8 [**(A)** T2-weighted MR image according to Rusbridge et al. (17) **(B)** T2-weighted MR image colored according to Rusbridge et al. (17)].

Figure S5 MR image cat 1 brain slice 8 [**(A)** T2-weighted MR image with tilted angle according to Milne et al. 10 **(B)** T2-weighted MR image with tilted angle colored according to Milne et al. (10)].

Figure S6 MR image cat 1 brain slice 8 [**(A)** FLAIR-weighted MR image according to Rusbridge et al. (17) **(B)** FLAIR-weighted MR image colored according to Rusbridge et al. (17)].
