# Supplementary figures and images for: Delineation of the Feline Hippocampal Formation: A Comparison of Magnetic Resonance Images With Anatomic Slices
Source: Front Vet Sci. 2019 Nov 8;6:358. doi: 10.3389/fvets.2019.00358 (PMC6857121; doi:10.3389/fvets.2019.00358)

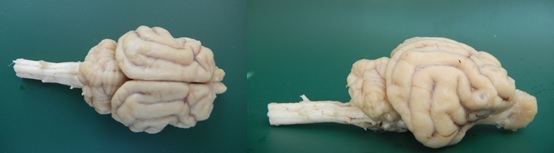

Supplement: Figure S1 — Brain after the formalin fixation. [file Image_1.JPEG]

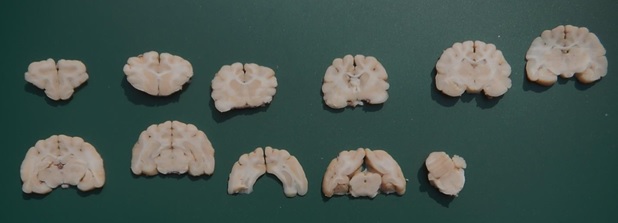

Supplement: Figure S2 — Overview over the produced slices. [file Image_2.JPEG]

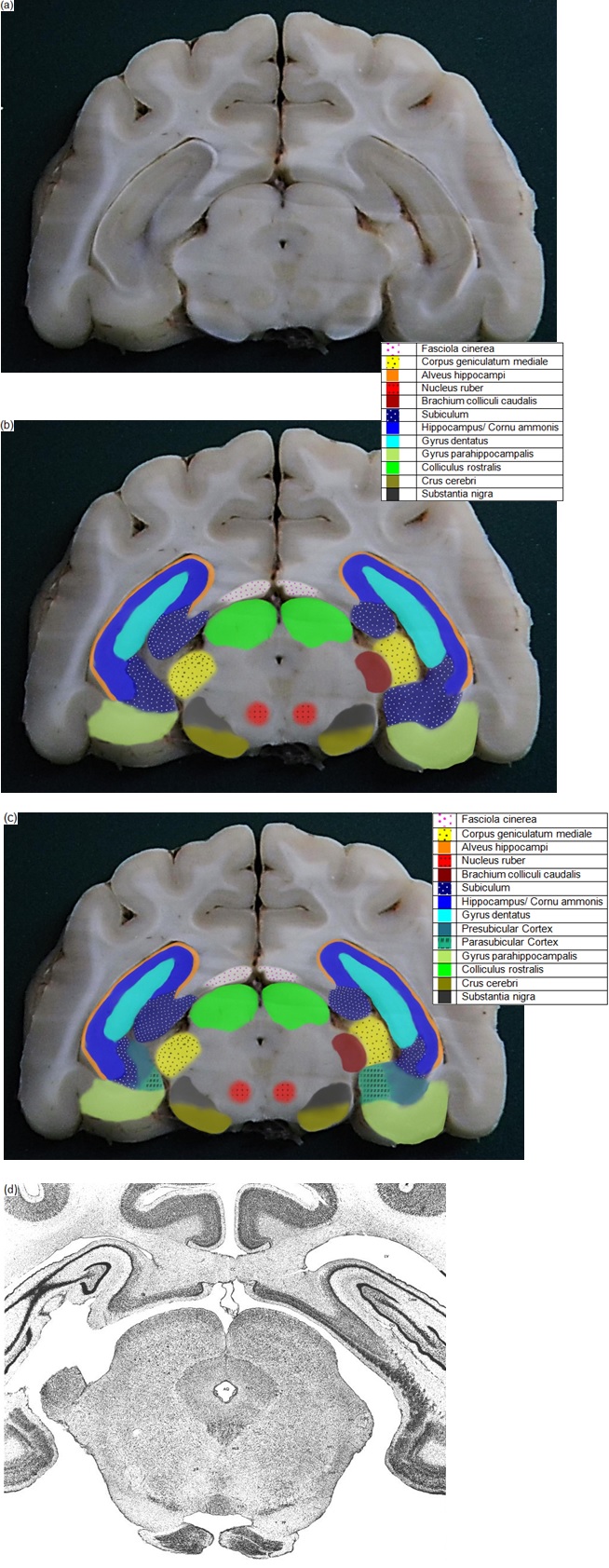

Supplement: Figure S3 — (A) Cat 1 slice 8 (B) colored (C) colored with pre- and parasubicular Cortex (D) Brainmaps (36). [file Image_3.JPEG]

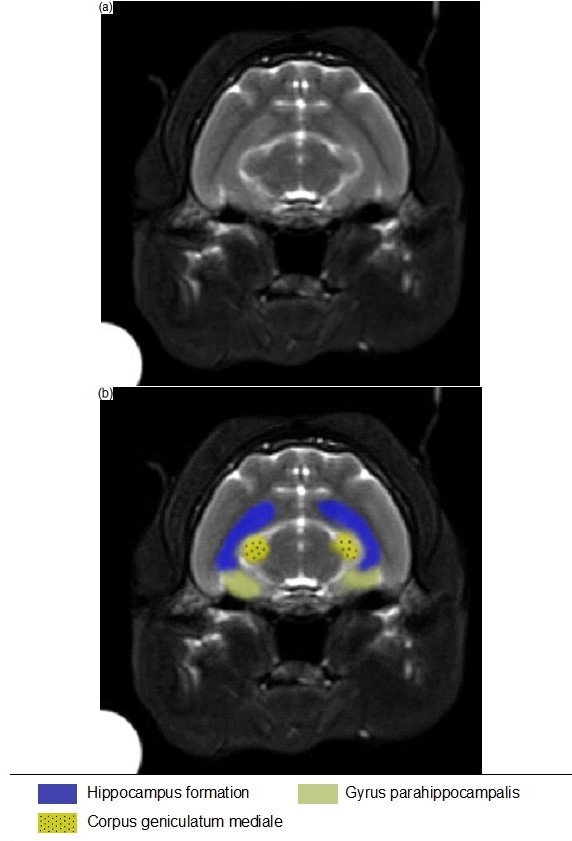

Supplement: Figure S4 — MR image cat 1 brain slice 8 [(A) T2-weighted MR image according to Rusbridge et al. (17) (B) T2-weighted MR image colored according to Rusbridge et al. (17)]. [file Image_4.jpeg]

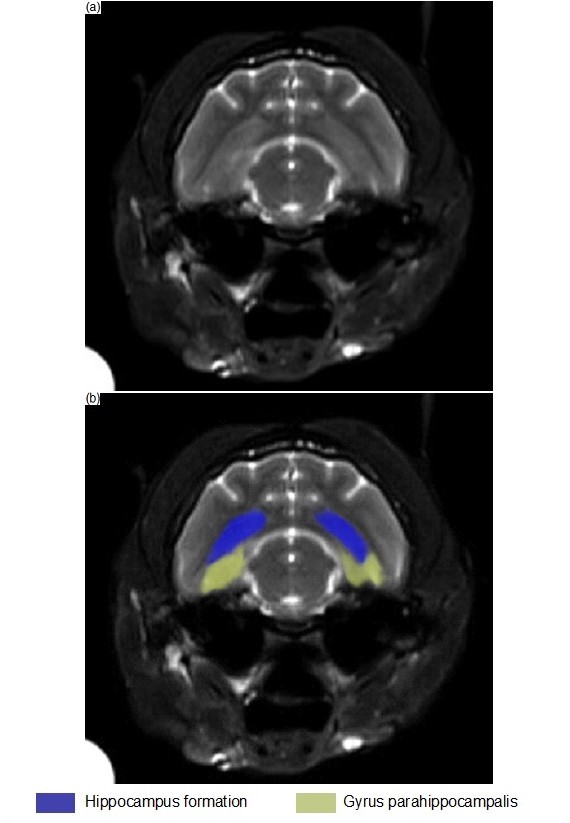

Supplement: Figure S5 — MR image cat 1 brain slice 8 [(A) T2-weighted MR image with tilted angle according to Milne et al. 10 (B) T2-weighted MR image with tilted angle colored according to Milne et al. (10)]. [file Image_5.jpeg]

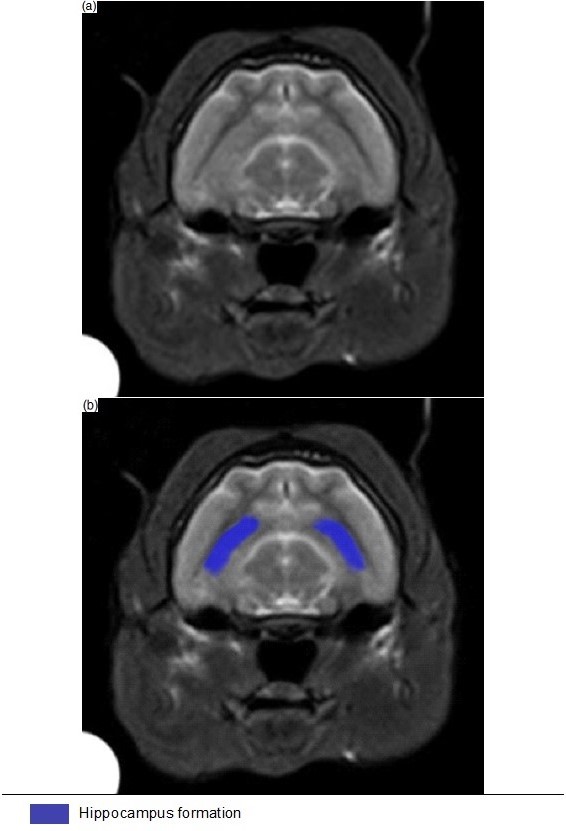

Supplement: Figure S6 — MR image cat 1 brain slice 8 [(A) FLAIR-weighted MR image according to Rusbridge et al. (17) (B) FLAIR-weighted MR image colored according to Rusbridge et al. (17)]. [file Image_6.jpeg]
